# Early intravenous unfractionated heparin and outcome in acute lung injury and acute respiratory distress syndrome – a retrospective propensity matched cohort study

**DOI:** 10.1186/1471-2466-12-43

**Published:** 2012-08-15

**Authors:** Jorrit J Hofstra, Alexander P J Vlaar, David J Prins, Gavin Koh, Marcel Levi, Marcus J Schultz, Jan M Binnekade, Nicole P Juffermans

**Affiliations:** 1 Department of Intensive Care Medicine, Academic Medical Center, University of Amsterdam, Meibergdreef 9, 1105 AZ, Amsterdam, The Netherlands; 2 Laboratory for Experimental Intensive Care and Anesthesiology (L.E.I.C.A.), Academic Medical Center, University of Amsterdam, Meibergdreef 9, 1105 AZ, Amsterdam, The Netherlands; 3 Department of Anesthesiology, Academic Medical Center, University of Amsterdam, Meibergdreef 9, 1105 AZ, Amsterdam, The Netherlands; 4 Department Internal Medicine of the Academic Medical Center, University of Amsterdam, Meibergdreef 9, 1105 AZ, Amsterdam, the Netherlands; 5 Department of Medicine, University of Cambridge, Cambridge, UK

**Keywords:** Acute lung injury, Heparin, Case–control study, Critical illness

## Abstract

**Background:**

Acute lung injury (ALI) is characterized by a pro-coagulant state. Heparin is an anticoagulant with anti-inflammatory properties. Unfractionated heparin has been found to be protective in experimental models of ALI. We hypothesized that an intravenous therapeutic dose of unfractionated heparin would favorably influence outcome of critically ill patients diagnosed with ALI.

**Methods:**

Patients admitted to the Intensive Care Unit (ICU) of a tertiary referral center in the Netherlands between November 2004 and October 2007 were screened. Patients who developed ALI (consensus definition) were included. In this cohort, the impact of heparin use on mortality was assessed by logistic regression analysis in a propensity matched case–control design.

**Results:**

Of 5,561 admitted patients, 2,138 patients had a length of stay > 48 hours, of whom 723 were diagnosed with ALI (34%), of whom 164 received intravenous heparin. In a propensity score adjusted logistic regression analysis, heparin use did not influence 28-day mortality (odds ratio 1.23 [confidence interval 95% 0.80–1.89], nor did it affect ICU length of stay.

**Conclusions:**

Administration of therapeutic doses of intravenous unfractionated heparin was not associated with reduced mortality in critically ill patients diagnosed with ALI. Heparin treatment did not increase transfusion requirements. These results may help in the design of prospective trials evaluating the use of heparin as adjunctive treatment for ALI.

## Background

Acute lung injury (ALI), and its more severe form Acute Respiratory Distress Syndrome (ARDS), are characterized by an exaggerated pulmonary pro-inflammatory and pro-coagulant response of the host against some form of insult. In sepsis, activation of coagulation as well as defective anticoagulant pathways and inhibition of fibrinolysis all contribute to systemic coagulopathy [1]. Similar disturbances in coagulation and fibrinolysis have been found locally in models of ALI [2-4] and in lungs of patients with ALI/ARDS [5,6]. There is evidence that this coagulopathy contributes to pulmonary inflammation [7,8], as the extent of coagulopathy is independently associated with adverse clinical outcomes in patients with ALI/ARDS [8,9]. As such, the pathogenesis of ALI/ARDS shares many similarities to sepsis [5,6] independent of the occurrence of sepsis [8]. Mechanical ventilation also induces fibrin deposition in the lung, thereby contributing to lung injury [8]. Infusion of recombinant human activated protein C (APC) was found to reduce mortality in patients with severe sepsis in a large phase III clinical trial [10], although this was offset by a recently finished trial of patients with septic shock [11]. A non-randomized subgroup analysis showed that APC was particularly effective in patients who presented with severe community–acquired pneumonia as the source of sepsis, although these findings were not confirmed in a clinical trial of sepsis patients with a low risk of death [12] or the recently finished trial of patients with septic shock [11]. The beneficial effect of APC in patients with severe sepsis observed in the PROWESS trial could, at least partially, be attributed to the effects of APC on lung coagulation [13,14]. A retrospective cohort study suggested that early administration of heparin is of benefit in patients diagnosed with septic shock [15], although this was not confirmed by a prospective clinical trial [16].

Studies evaluating the effect of anticoagulant therapy in ALI, mostly in experimental settings, are promising [17,18]. Animal studies have demonstrated that administration of heparins, APC, antithrombin (AT), tissue factor–factor VIIa (TF–FVIIa) pathway inhibitors, plasminogen activators and thrombomodulin can attenuate pulmonary coagulopathy, reduce lung injury and/or improve oxygenation [19].

Besides its anticoagulant effects, unfractionated heparin has been shown to have a wide range of anti-inflammatory and immunomodulatory effects [20-22]. Infusion of heparin was found to limit lung injury in endotoxemic swine [23] and mice [24] and reduced pulmonary fibrin depositions in a combined model of smoke inhalation and lung injury in sheep, thereby improving oxygenation [25]. In a rat model of acute lung injury, heparin was found to have positive effects through inhibiting nitric oxide synthase [26]. Further, low molecular weight heparin reduced hyperoxia-induced lung injury in mice through protein kinase interactions [27]. In a clinical study, nebulized heparin was found to significantly reduce activation of coagulation in the lungs of ALI patients [28], further underlining the hypothesis that unfractionated heparin could favorably influence outcome of ALI.

A significant subset of critically ill patients with ALI/ARDS receives heparin early after admission to the ICU [15]. Indications for heparin include respiratory insufficiency due to myocardial infarction, (suspected) venous thromboembolism, atrial fibrillation and coumarin derivate treatment prior to ICU admission. Another indication for heparin treatment is the use of renal replacement therapy. Given the anticoagulant and anti-inflammatory properties of heparin, we hypothesized that early heparin is associated with reduced mortality among patients diagnosed with ALI/ARDS. Therefore, we evaluated the impact of systemically administered therapeutic dose of heparin on outcome in ALI patients in terms of mortality as well as length of ICU stay.

## Methods

### Design

This is a nested case control study design, derived from a retrospective cohort of ALI/ARDS patients [29]. The study was approved by the Medical Ethics Committee of the Academic Medical Center, Amsterdam, The Netherlands (reference 08.17.0964), who waived the requirement for individual informed consent in view of the retrospective nature of this research.

### Patient population

Using an electronic patient data monitoring system, all patients with a first admission to the mixed medical-surgical Intensive Care ward of a tertiary referral center in the Netherlands from 1 November 2004 until 1 October 2007 were reviewed and screened. Patients who developed ALI (consensus definition) were included. In order to determine the effect of heparin on patients who develop ALI while being on the ICU, patients with a length of stay shorter than 48 hours were excluded.

ALI was defined using the consensus definition of ALI: new onset hypoxemia or deterioration, demonstrated by a PaO_2_/FiO_2_ < 300 mmHg, with bilateral pulmonary changes, in the absence of cardiogenic pulmonary edema [30]. Pulmonary edema was diagnosed as being of cardiogenic origin if the pulmonary arterial occlusion pressure was >18 mmHg. In the absence of pulmonary artery wedge catheter measurements, cardiac failure was diagnosed if two of the following were present: central venous pressure >15 mmHg, a history of heart failure or valve dysfunction, ejection fraction <45% as estimated by echocardiogram, or a positive fluid balance. The probability of cardiogenic pulmonary edema was scored by two physicians independently on a scale of 1–4 (APJV and NPJ) [31]. Chest radiographs were scored for the presence of new onset bilateral interstitial abnormalities by two independent physicians who were blinded to the predictor variables. When interpretation differed, chest radiograph and the description by the radiologist were reviewed and discussed until consensus was reached. Use of heparin was scored when patients received heparin within 48 hours of diagnosis of AL/ARDS.

### Patient data collection

All data was collected from our hospital patient database. We collected the APACHE II score, length of ICU stay and mortality. Potential ALI risk factors were scored as positive when present 48 hours prior to onset of ALI, including trauma, elective and emergency surgery, lung contusion, aspiration, massive transfusion, pancreatitis, pneumonia, sepsis and mechanical ventilation. Life support measures requiring the use of inotropic or vasopressor support were also scored.

Co-interventions that may influence outcome of ALI were scored, including mechanical ventilation with tidal volumes ≤ 8 ml/kg [32], restrictive fluid strategy (net fluid balance in the first 7 days < 0 ml) [33], use of APC [11] and use of steroids (>300 mg hydrocortisone per day or the equivalent) [34]. Co-morbidities that influence use of heparin were scored, including myocardial infarction, surgery and hematologic malignancy. Laboratory parameters scored on admission included platelet count, prothrombin time (PT) and activated partial thrombin time (aPTT).

### Outcome measures

The primary outcome measure was mortality over 28 days. Secondary outcome variables included 90 day mortality. Mortality data was cross-checked with the Dutch Civil Registry. Safety of heparin administration was assessed by comparing the need for allogeneic blood transfusion.

### Statistical analysis

Continuous variables are expressed as mean and standard deviation or medians and interquartile ranges depending on distribution. Categorical variables are expressed as n (%). Comparison between groups was done with Student’s *t-*test or Mann–Whitney *U* test depending on distribution. Categorical variables were compared with the Chi-square test or Fisher’s exact test.

Cases were defined as patients who died within 28 days of admission to the ICU. Cases were matched to controls using propensity analysis [35,36]. Propensity score was calculated by a multivariable logistic regression model with heparin as the dependent variable, while all other covariates related to mortality were included as independent variables. Covariates included age, sex, APACHE II score, pre-existing medical condition (trauma, sepsis, aspiration, pancreatitis, pneumonia, massive transfusion), recent surgical history, respiratory failure requiring mechanical ventilation, cardiovascular failure requiring inotropic/vasopressor support and co-interventions for ALI (use of APC or steroids, limited tidal volume ventilation, restrictive fluid strategy). The joint probability of these covariates to predict heparin use is used to match cases with controls in a 1 to 3 fashion, to control for confounding and bias due to non-random selection of patients. We considered there might be a causal relation between heparin treatment and APACHE II score as both may occur within the 24 hours following admission, therefore we calculated propensity scores both with and without using the APACHE II score in our model.

Heparin use related to 28 days mortality as well as to 90 days mortality was investigated using conditional logistic regression. As effects of heparin may actually reflect effects related to a prolonged aPTT, comparisons were repeated between patients with an aPTT of 45 seconds or more and an aPTT of less than 45 seconds, using a mixed ANOVA model for repeated measures (4 measurements). Furthermore, we used a Cox proportional hazards model with 28-day mortality as the dependant variable and heparin use and propensity scores as covariates, in order to estimate the relative impact of heparin use on 28-day all-cause mortality.

Statistical analysis was conducted with SPSS version 16.0 (SPSS Inc., Chicago, IL, USA).

## Results

### Patient populations

**Figure 1:**
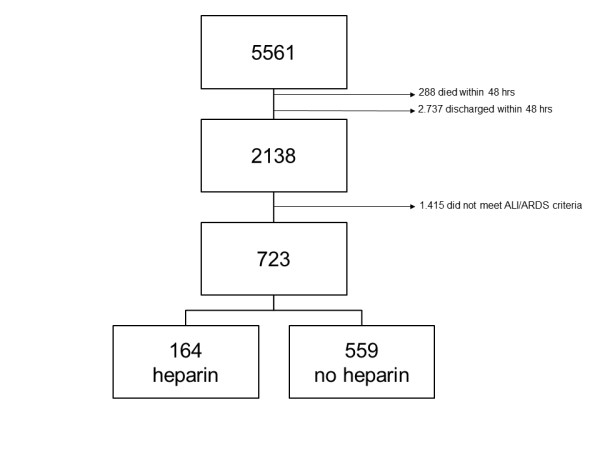
**Flowchart of patients diagnosed with acute lung injury.** Abbreviations; ALI = acute lung injury, ARDS = acute respiratory distress syndrome.

During the screening period, 5,561 patients were admitted to our ICU. Of these, 398 patients were re-admissions, 288 died within 48 hours and 2,737 were discharged from the ICU within 48 hours, leaving 2,138 patients for screening of the presence of ALI (Figure 1). Of these, 1,415 patients did not meet ALI criteria, leaving 723 patients for analysis. The inter observer agreement for the diagnosis of ALI was good (weighted kappa 0.61).

Of the 723 patients, 164 patients received therapeutic dose intravenous heparin. Baseline characteristics of the (unmatched) patients are shown in Table 1. Outcome did not differ between unmatched patient groups. Groups were unbalanced with respect to age, APACHE II score and type of admission diagnosis.

**Table 1:** Patient characteristics and outcome of unmatched ALI patients

	**No Heparin n = 559**	**Heparin n = 164**	***p*value**
Age, mean (SD)	59 (17)	63 (14)	0.003
Male,% (n)	70 (389)	63 (103)	0.10
APACHE II score, mean (SD)	17.8 (6.9)	19.8 (6.5)	0.001
Medical patients,% (n)	60 (334)	71 (117)	0.007
Surgery elective,% (n)	23 (128)	19 (31)	0.28
Surgery emergency,% (n)	17 (97)	7 (16)	0.02
Medical condition			
Multiple Trauma,% (n)	8 (43)	2 (3)	0.007
Sepsis,% (n)	21 (115)	18 (29)	0.42
Diabetes,% (n)	14 (81)	16 (27)	0.53
Liver Failure,% (n)	3 (14)	2 (4)	0.96
Hematological malignancy,% (n)	6 (35)	2 (3)	0.03
Aspiration,% (n)	5 (26)	4 (6)	0.59
Pancreatitis,% (n)	2 (12)	2 (4)	0.82
Pneumonia,% (n)	23 (129)	10 (17)	< 0.001
COPD	11 (62)	14 (23)	0.31
Auto immune disease,% (n)	6 (34)	9 (15)	0.17
Previous Myocardial infarction,% (n)	13 (72)	40 (66)	< 0.001
Immune Compromised,% (n)	11 (61)	6 (10)	0.07
Massive Transfusion,% (n)	14 (78)	12 (19)	0.43
Life support measures			
Respiratory failure (MV) ,% (n)	93 (519)	95 (156)	0.42
Cardiovascular failure (MI) ,% (n)	13 (72)	40 (66)	<0.001
Co interventions			
Activated Protein C,% (n)	4 (24)	1 (1)	0.02
Stress dose steroids,% (n)	42 (233)	46 (76)	0.29
Protective mechanical ventilation (<8 ml/kg) ,% (n)	54 (304)	55 (91)	0.29
Restrictive fluid strategy (net fluid balance first 7 days ≤ 0 ml) ,% (n)	36 (203)	35 (58)	0.82
Outcome			
28 days mortality,% (n)	24 (134)	30 (50)	0.09
90 days mortality,% (n)	32 (181)	39 (64)	0.11
ICU-stay, median (IQR)	7 (4–13)	7 (4–13)	0.59
Hospital-stay, median (IQR)	20 (11–38)	18 (8–37)	0.13

*APACHE* = Acute Physiology and Chronic Health Evaluation, *COPD* = chronic obstructive pulmonary disease, *IQR* = interquartile range, *MI* = myocardial infarction, *MV* = mechanical ventilation, *SD* = standard deviation.

### Propensity matching

Using logistic regression analysis, risk factors for 28-day mortality were determined, shown in Table 2. As expected, old age and high APACHE II scores were risk factors for mortality in ALI patients, whereas undergoing elective surgery protected against mortality. Diabetes, liver failure, autoimmune disease and a compromised immune state were risk factors for mortality in this cohort. Of interest, cardiovascular failure did not contribute to mortality in ALI patients. Application of limited tidal volume ventilation and the restrictive use of fluids were confirmed as protective measures in this cohort of ALI patient. Interestingly, a stress dose steroid regimen contributed to mortality in this cohort of ALI patients. The covariates that significantly contributed to outcome (including APACHE II score) were used in a propensity-based analysis of the risk to die of patients who had received heparin and controls, which is shown in Table 3. Suitable propensity matches were found for all patients receiving heparin. Use of heparin did not alter mortality risk compared to controls.

**Table 2:** Multivariate logistic regression analysis of covariates for the risk of 28-day mortality in ALI patients

	**OR (95% CI)**	*P *value*
Age	1.02 (1.00/1.03)	0.002
Male	1.39 (0.92/1.86)	0.13
APACHE II score	1.09 (1.06/1.12)	< 0.001
Medical	1.94 (1.34/2.81)	< 0.001
Surgery Elective	0.40 (0.25/0.65)	< 0.001
Surgery Emergency	0.91 (0.57/1.45)	0.68
Medical conditions		
Multiple Trauma	0.51 (0.22/1.15)	0.11
Sepsis	1.27 (0.84/1.90)	0.25
Diabetes	1.83 (1.18/2.83)	0.006
Liver Failure	3.82 (1.48/9.82)	0.006
Hematological malignancy	1.77 (0.89/3.49)	0.10
Aspiration	1.35 (0.63/2.91)	0.44
Pancreatitis	0.67 (0.19/2.38)	0.54
Pneumonia	1.14 (0.75/1.71)	0.55
COPD	1.26 (0.76/2.07)	0.37
Auto immune disease	1.95 (1.07/3.56)	0.03
Previous Myocardial infarction	1.25 (0.83/1.89)	0.29
Immune Compromised	1.69 (1.00/2.83)	0.05
Massive Transfusion	0.68 (0.40/1.16)	0.16
Life support measures		
Respiratory failure at admission	1.00 (0.98/1.02)	0.99
Cardiovascular failure (inotropic at admission)	1.00 (0.98/1.02)	0.84
Co interventions		
Activated Protein C	1.40 (0.59/3.29)	0.45
Stress dose steroids	1.89 (1.34/2.64)	< 0.001
Protective mechanical ventilation (<8 ml/kg)	0.63 (0.45/0.89)	0.008
Restrictive fluid strategy (net fluid balance first 7 days ≤ 0 ml)	0.33 (0.22/0.49)	< 0.001

* Potentially confounder (*p* value ≤ 0.10). *COPD* = chronic obstructive pulmonary disease, *OR* = odds ratio, *CI* = 95% confidence limit.

**Table 3:** Conditional logistic regression of mortality of patients with acute lung injury matched using propensity score

	**Mortality rate (No. deaths/total No. of patients)**	**OR (95% CI)**	***p***
Heparin use^1^ (28-day mortality)	30 (50/164)	1.22 (0.79/1.90)	0.36
Heparin use^1^ (90-day mortality)	39 (64/164)	1.08 (0.73/1.59)	0.71

^1^ Matched with propensity score.

### Primary and secondary outcome

The length of ICU stay was also not different in the heparin group compared to the control group, p 0.59 (Table 1). A repeated analysis leaving out the APACHE II score yielded similar results. Heparin use did not influence mortality, as it affected neither 28-day (1.23 (0.80/1.89) p = 0.34) nor 90-day mortality (1.27 (0.85/1.89) p = 0.24).

As any possible protective effect of heparin on the occurrence of ALI may be due to dose, reflected by prolongation of the activated partial thromboplastin time (aPTT), odds ratio’s for mortality were recalculated stratified for aPTT (Table 4). In all ALI patients and in those using heparin, a prolonged aPTT did not protect against mortality. In patients not receiving heparin, an aPTT < 45 seconds was found to protect against mortality.

**Table 4:** Mortality over 28 days in patients with acute lung injury stratified for aPTT

	**Mortality rate (No. deaths/total No. of patients)**	**OR (95% CI)**	***p***
aPTT ≤ 45 (all patients)	25.4 (184/723)	0.64 (0.43/.96)	0.03
aPTT ≤ 45^1^ (patients using heparin)	31 (50/164)	0.66 (0.30/1.45)	0.26
aPTT ≤ 45^2^ (patients not using heparin)	24 (134/559)	0.54 (0.34/0.87)	0.007
Pooled results ^1,2^		0.57 (0.38/0.86)	0.005

**Figure 2:**
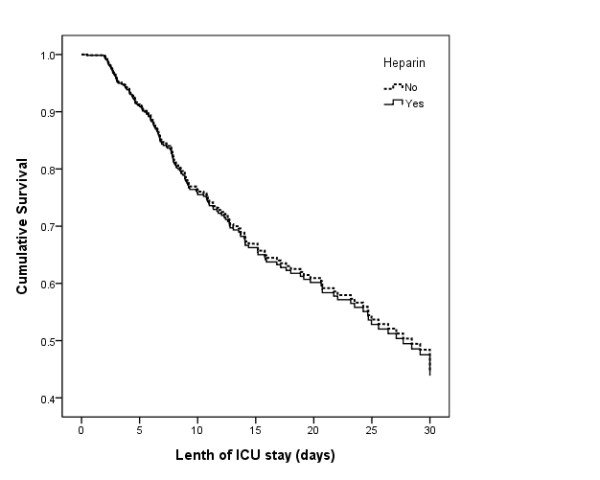
Cumulative survival of patients receiving heparin vs. patients who had not received heparin based on a Cox proportional hazard model adjusted for propensity.

In an unadjusted Cox model, the risk of mortality did not differ in patients who had received heparin compared to patients who had not received heparin [hazard ratio (HR) 0.97; 95% CI 0.67–1.40; p 0.87]. Adjustment for propensity score did not alter risk of mortality (adjusted HR 0.82; 95% CI 0.57–1.19; p 0.30). Figure 2 expresses cumulative survival in both groups using this model.

The need for allogeneic blood transfusion during ICU stay did not differ between the heparin-treated group and the control group (44% vs. 47%, p = 0.3), nor the mean amount of red blood cells units transfused (0.9 ± 0.8 vs. 0.9 ± 1.0, p = 0.5).

## Discussion

In this retrospective, propensity matched case control study, the use of a therapeutic dose of unfractionated heparin was not associated with a decrease in mortality in critically ill patients with ALI. Based on these results, heparin does not seem to benefit patients with ALI.

These results are in contrast with previous experimental reports suggesting that intravenous heparin favorably influences outcome in ALI [23,37,38] and also with a prospective study, in which nebulized heparin significantly reduced coagulation activation in the lungs of critically ill ALI patients [28] and shortened duration of mechanical ventilation [39].

Our study has some important limitations. First and foremost, sample size may have been too small to demonstrate a statistically significant impact on mortality. The study was not designed to look for an effect of heparin on ALI and may have been underpowered. Indeed, interventions studied in randomized trials with a proven effect on outcome in ALI have required a large number of patients [32,33]. We were not able to measure markers of inflammation in the groups and cannot comment on the ability of intravenous heparin to influence inflammation in ALI.

Second, the retrospective nature of the study design is subject to bias. Indeed, groups differed in several baseline characteristics. In an effort to limit confounding, propensity analysis was performed, which resulted in elimination of these baseline differences. Although retrospective analyses cannot replace the advantage of randomization, propensity analyses may be a way of reducing bias when assessing treatment effects [36]. It is possible that some patients were already receiving heparin upon ICU admission. Since APACHE II score is calculated in the first 24 hours of ICU admission, one could question the justification of using APACHE II score when calculating propensity for heparin. However, a repeat analysis without APACHE II score resulted in similar findings. However, this method may still allow for confounders unaccounted for. Another possible baseline difference that could not be accounted for in the propensity analysis is the dose of heparin used, as heparin treatment regimen was not standardized in this retrospective analysis. Indications for the use of heparin were not scored and heparin dose in our ICU differs depending on indication (e.g., suspected thrombo-embolism or anticoagulant requirement due to use of renal replacement therapy). Therefore, analyses were repeated according to aPTT. Prolonged aPTT also did not yield protection in ALI patients. Another explanation could be the low levels of pulmonary antithrombin (AT). The anticoagulant effects of heparin are exerted via AT, and it may well be that AT consumption leads to impaired actions of heparin. Previous animal studies have demonstrated that AT combined with heparin effectively reduces acute lung injury [40].

Third, as the focus of inflammation in ALI patients is in the lungs and nebulized heparin was protective in ALI [28,39], it may be possible that the use of nebulized heparin instead of intravenous heparin would have yielded an effect on outcome in this study, as found before [38,41]. We cannot comment on this issue, as the intravenous route is currently standard clinical practice.

Fourth, a potential benefit of the use of heparin on outcome may have been offset by complications of the use of heparin, in particular bleeding. Although we were unable to score bleeding in this retrospective analysis, it seems unlikely that bleeding influenced mortality in this study, given that groups did not differ in their need for allogeneic blood transfusion.

## Conclusions

In conclusion, administration of therapeutic doses of intravenous unfractionated heparin was not associated with reduced mortality in critically ill patients diagnosed with ALI. Heparin treatment did not increase (bleeding) complications.

### Future investigations

In view of the limitations of our study design and the abundant data from previous (pre-) clinical investigations, a prospective clinical trial is warranted to investigate the potential of heparin treatment in critically ill patients diagnosed with ALI. Since coagulopathy in acute lung injury is for the most part restricted to the lung, future investigations may include local treatment allowing increased local efficacy while reducing the risk on systemic bleeding.

## Abbreviations

rh–APC, Recombinant human activated protein C; ALI, Acute lung injury; ARDS, Acute respiratory distress syndrome; aPTT, Activated partial thrombin time; APACHE II, Acute Physiology And Chronic Health Evaluation II; AT, Antithrombin; CI, Confidence interval; ICU, Intensive care unit; OR, Odds ratio; PT, Prothrombin time.

## Competing interests

The author(s) declare that they have no competing interests.

## Authors’ contributions

JJH participated in the design of the study, participated in collecting and analyzing the data, participated in the conduction of statistical analysis and participated in drafting of the manuscript. APV participated in collecting and analyzing the data and participated in drafting of the manuscript. DP participated in collecting and analyzing the data and participated in drafting of the manuscript. GK participated in collecting and analyzing the data and participated in drafting of the manuscript. ML participated in the design of the study and participated in drafting of the manuscript. MJS participated in the design of the study and participated in drafting of the manuscript. JMB coordinated in the conduction of statistical analysis and participated in drafting of the manuscript. NPJ participated in the design of the study, participated in collecting and analyzing the data, participated in the conduction of statistical analysis and participated in drafting of the manuscript. All authors have read and approved the final manuscript.

## Pre-publication history

The pre-publication history for this paper can be accessed here:

http://www.biomedcentral.com/1471-2466/12/43/prepub
